# 

**Published:** 1885-10

**Authors:** 


ORIGINAL COMMUNICATIONS.—Eulogy on Count Ercolani. Elmer R. Reynolds, M.D........ 297
Acute Parenchymatous Nephritis A. W. Clement, V.S........................................ 308
Clinical Aspect of some Disorders of the Gastro Intestinal Tract of the Horse. Thomas B. Rogers, V.S........................................ 315
An American Veterinarian's Impressions of the Profession in England. Austin Peters, D.V.S., M.R.C.V.S........................................ 322
Experience with Mammitis. Chas. A. Meyer, D.V.S........................................ 331
Human and Bovine Tuberculosis. J. M. Heard, M.R.C.V.S........................................ 333
The Inoculation of Hogs, with Reference to Erysipelas. Prof. Dr. Schutz........................ 335
Emmerich's so-called "Naples Cholera Bacteria."........................................ 342
EDITORIAL.—The Necessity of an Organized Inspection of our Meat and Milk supply by Educated Veterinarians.—None too Much Quarantine—State Veterinarians.—Inoculation Against Black-leg.—The duty on Scientific Books and Instruments.—Report of the Central Veterinary School at Munich, Bavaria, for the years 1883-84.—Annual Report of the Royal Imperial Veterinary School, Vienna, Austria, 1883-84.—Segar in the Urine of Cows in Paturient Apoplexia.—The U. S. Veterinary Medical Association........................................ 348
CASE DEPARTMENT.—Autopsies at Central Park Menagerie........................................ 361
Laceration of the Flexor Metatarsi........................................ 363
PROCEEDINGS OF VETERINARY MEDICAL SOCIETIES.—N. Y. State cademy of Veterinary Science and Comparative Pathology.—Massachusetts Veterinary Association.—Veterinary Medical Association of New Jersey........................................ 364
CORRESPONDENCE........................................ 371
OBITUARY........................................ 372
REVIEWS........................................ 373

